# Stimulation with Concanavalin-A Induces IL-17 Production by Canine Peripheral T Cells

**DOI:** 10.3390/vetsci2020043

**Published:** 2015-04-10

**Authors:** Michelle G. Ritt, Beth A. Lindborg, Timothy D. O’Brien, Joseph Bisignano, Jaime F. Modiano

**Affiliations:** 1 Department of Veterinary Clinical Sciences, College of Veterinary Medicine, University of Minnesota, St. Paul, MN 55108, USA; E-Mails: j.bisignano@yahoo.com (J.B.); modiano@umn.edu (J.F.M.); 2 Department of Veterinary Population Medicine, College of Veterinary Medicine, University of Minnesota, St. Paul, MN 55108, USA; E-Mails: ziem0052@umn.edu (B.A.L.); obrie004@umn.edu (T.D.O.B.); 3 Stem Cell Institute, University of Minnesota, Minneapolis, MN 55455, USA; 4 Masonic Cancer Center, University of Minnesota, Minneapolis, MN 55455, USA; 5 Center for Immunology, University of Minnesota, Minneapolis, MN 55455, USA

**Keywords:** canine, cytokine, flow cytometry, T Lymphocytes

## Abstract

The characteristics of canine IL-17-producing cells are incompletely understood. Expression of mRNA encoding orthologs of IL-17 and the IL-17 receptor has been documented in tissues from dogs with arthritis, inflammatory bowel disease, and lymphoma; however, no associations have been found between IL-17 gene expression and disease phenotype in these conditions. Robust assessment of the role of IL-17-producing cells in dogs will require measuring the frequency of these cells in health and disease in balance with other lymphocyte subsets. The aim of this study was to confirm that the T-cell IL-17 response in dogs is evolutionarily conserved. Canine peripheral blood mononuclear cells were stimulated with Concanavalin A with or without polarizing cytokines. We used a canine specific IL-17 ELISA and flow cytometry to identify IL-17-producing T cells. Accumulation of intracellular IL-17 was observed in stimulated CD4 and CD8 T cells. The addition of pro-inflammatory cytokines appeared to enhance polarization of canine CD4 T cells to the Th17 phenotype. Conversely, the addition of IL-2 in the presence of TGF-β resulted in expansion of Treg cells. We conclude that canine IL-17-producing cells behave similarly to those from humans and mice when stimulated with mitogens and polarized with pro-inflammatory or immune regulatory cytokines.

## 1. Introduction

Interleukin-17 (IL-17) is a pro-inflammatory cytokine produced by a subset of T helper cells (Th17). In humans and rodents, these cells act as physiological mediators of inflammation that provide an important counterbalance to the suppressive effects of regulatory T cells (Tregs) [1]. In both of these species, Th17 expansion is favored in the presence of IL-6, IL-1β and transforming growth factor-β (TGF-β), whereas Treg expansion is favored in the presence of IL-2 and TGF-β [1,2]. Excessive and/or inappropriate Th17 responses have been associated with various immune-mediated diseases [3]. However, the identity and function of Th17 cells in dogs and their role in canine autoimmune diseases have yet to be precisely determined [4]. This is at least partly due to the lack of validated reagents to characterize these cells functionally and phenotypically.

IL-17 production is not restricted to Th17 cells. This cytokine also can be produced by a subset of CD8 T cells (Tc17) in humans and mice [5], as well as by a subset of MHC class II-restricted, CD4/CD8 double negative (DN) T cells, at least in mice [6]. Here, we used flow cytometry and an enzyme-linked immunosorbent assay (ELISA) to assess IL-17 production by mitogen-activated canine peripheral blood mononuclear cells (PBMC) and to determine the effect of cytokine polarization on the generation of Th17 and Treg cells. Our results show that IL-17 production is an evolutionarily conserved process that follows predictable patterns upon T-cell activation.

## 2. Results and Discussion

### 2.1. Mitogen-Stimulation Promotes IL-17 Production by Canine CD4 and CD8 T Cells

The presence of IL-17 producing cells in dogs was previously inferred from detection of IL-17 mRNA using quantitative real-time reverse-transcriptase polymerase chain reaction or gene expression microarrays [4,7,8,9]. However, to our knowledge cell-associated expression of canine IL-17 protein and enumeration of IL-17-producing canine T cells have yet to be determined. Thus, we first examined the capability to detect canine IL-17-producing T cells in culture after stimulation by Concanavalin A (ConA) [10,11].

**Figure 1:**
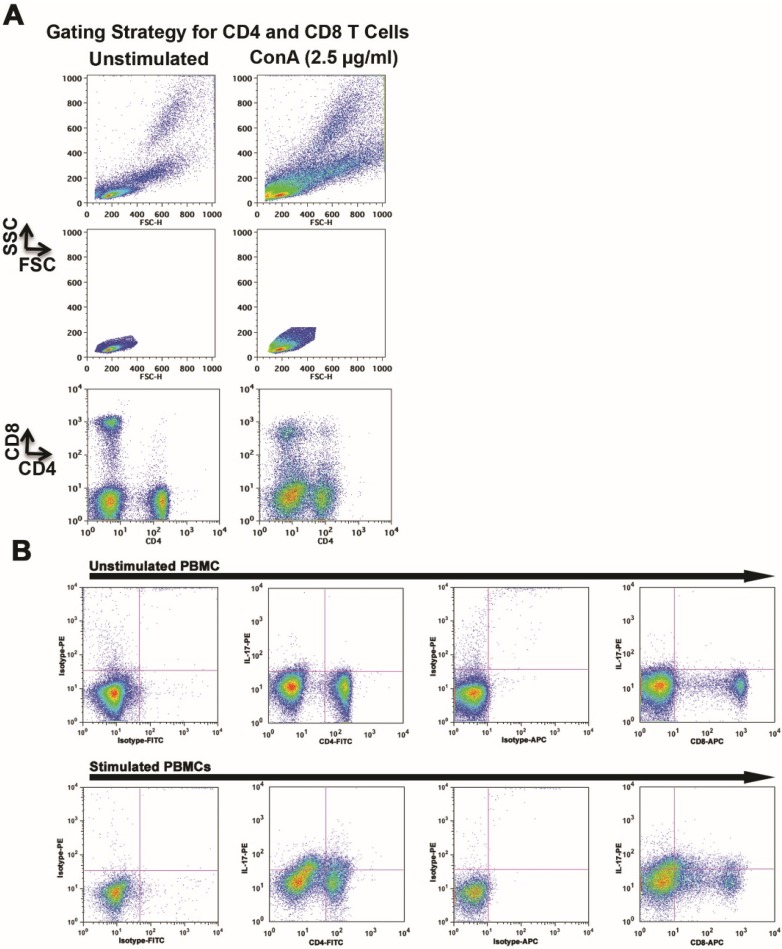
Stimulation of canine peripheral T cells promotes IL-17 production. (**A**) Peripheral T cells were gated from unstimulated (left) or ConA-stimulated (right) PBMCs using light scatter properties to define lymphocyte regions. The top panel shows ungated PBMCs and the middle panel shows the lymphocyte population after the white blood cell population gates were defined. The bottom panel shows separation of CD4^+^ and CD8^+^ cells based on immunostaining. (**B**) IL-17 expression was determined by intracellular staining and flow cytometry in the CD4^+^ and CD8^+^ subsets from ***a***. Two-dimensional dot plots show staining with isotype control antibodies, as well as staining of CD4 and CD8 cells with anti-IL-17 antibody in unstimulated (top) and ConA-stimulated (bottom) cells. These results represent Dog 1 shown in Table 1. All dogs tested showed expression of IL-17 in at least one T cell subset (see Table 1).

Figure 1A shows the gating strategy used to evaluate intracellular IL-17 in CD4 and CD8 cells from one of four canine PBMC samples (top left panels). The first region (Figure 1A) was created from two-dimensional forward angle (FSC) and right angle (SSC) light scatter dot plots to include cells with properties for lymphocytes and lymphoblasts (middle left panels). CD4 and CD8 subsets were then identified as shown in the bottom left panels and each population was analyzed for intracellular IL-17. Figure 1B shows accumulation of IL-17 was detectable in both CD4 and CD8 T-cells after ConA stimulation. There was a unimodal shift in the mean fluorescence intensity (MFI) in IL-17-stained samples as compared to isotype controls. This shift was consistently greater in the ConA-stimulated population than in the unstimulated population, but in addition, IL-17 staining in these ConA-stimulated populations included a “shoulder” of bright events, likely representing those cells responsible for most of the IL-17 production. This was also observed when CD4^+^, CD8^+^, and CD4^−^/CD8^−^ populations were analyzed individually, with an approximate two-fold increase in the MFI observed in CD4+ and in CD8^+^ cells as compared to CD4^−^/CD8^−^ cells.

**Table 1:** Percentage of IL-17^+^ CD4 and CD8 cells after stimulation with ConA for 6 h ^a^.

	%IL-17-Producing Cells in ConA Blasts (6 h)	
Dog #	CD4	CD8
1	12.4	17.6
2	4.5	4.8
3	11	5.2
4	1.0	8.8

^a^ PBMCs from four dogs were stimulated as described in Figure 1. The same gating strategy was used, except that the frequency of IL-17 “bright” events was verified in IL-17 (FL2) histograms where the percent positive cells for each population of stimulated cells was determined by considering the top 0.5% of the unimodal control (unstimulated cells) in each dog as the minimum limit for gating.

Table 1 shows a summary of the percentage of IL-17-bright CD4 and CD8 T cells from four different dogs. The demonstrable, albeit modest increase in IL-17-producing cells after stimulation of PBMC is consistent with data obtained after activation of human cells [10]. Tregs (FoxP3^+^/CD25^+^) in unstimulated and stimulated CD4 populations also were quantified for each dog. The percent Treg cells was variable, comprising 0.4%–12% of all CD4 cells. Treg cells showed no change after stimulation in two dogs (2% and 5%, of the CD4^+^ populations, respectively), a decrease in one dog (4.1% to 0.4%), and an increase in one dog (5.1% to 12%).

### 2.2. Pro-Inflammatory Cytokines Promote Polarization of Canine T Helper Cells towards the Th17 Phenotype

Experiments using T cells from humans and mice have shown that adding TGF-β, IL-1β, and IL-6 to the stimulation conditions facilitated development of Th17 cells, whereas including TGF-β and IL-2 led to expansion of Tregs [1,2]. To determine if this also occurred in dogs, we cultured canine PBMCs in the presence or absence of ConA as above. After three days, fresh media was replenished to the unstimulated cells and ConA-stimulated cells were divided into groups that received media alone, media supplemented with IL-2 and TGF-β, or media supplemented with IL-1β, IL-6, and TGF-β.

**Figure 2:**
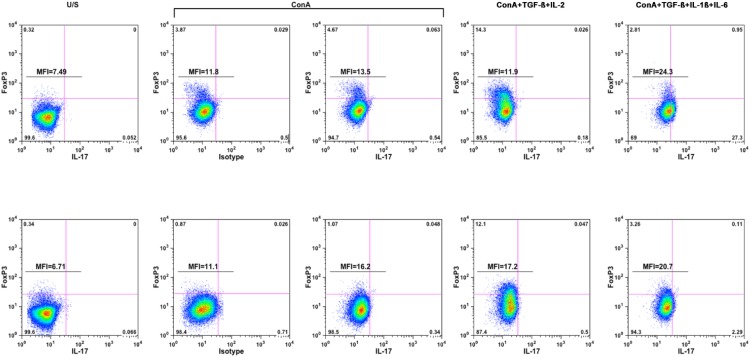
Addition of TGF-β, IL-1β, and IL-6 increases polarization of CD4^+^ cells toward the Th17 phenotype. PBMCs from two dogs were stimulated as described in the main text and analyzed for polarization of T helper (CD4^+^) cells toward FoxP3^+^ (Treg) and IL-17^+^ (Th17) phenotypes using flow cytometry. CD4^+^ cells were identified as described in Figure 1, and stimulation conditions are shown above the top panels, with isotype control staining shown for comparison in the ConA-stimulated samples. Gating was done using isotype control staining for stimulated cells, and MFI was calculated for all CD4^+^ T cells stained with IL-17 or with the matched isotype control (FL2). The MFI value for unstained cells in both dogs was 5.5.

We examined if the effects of polarization were evident using intracellular staining as above. For this, we gated CD4 T cells and evaluated expression of FoxP3 protein and accumulation of intracellular IL-17 to establish the existence of a canine Th17 subset. Figure 2 shows the frequency of IL-17-producing cells and Treg (FoxP3^+^) cells in the CD4 population from two healthy dogs. The data show modest Treg expansion in five-day ConA-stimulated T cells (compare the U/S and ConA panels) that was further enhanced by addition of IL-2 and TGF-β, but not by addition of IL-1β, IL-6, and TGF-β. Expansion of cells producing IL-17 at this point in five-day ConA-stimulated cells was also modest, with a small shift in MFI similar to that observed in cells during the initial phase of stimulation (Figure 1). The frequency of IL-17-producing CD4 T cells was unaffected by addition of IL-2 and TGF-β, but predictably, addition of IL-1β, IL-6, and TGF-β increased both the MFI of the CD4 T cell population and the frequency of IL-17 “bright” events.

Generally, these are expected results that mirror what has been shown in humans and mice [12], supporting the evolutionary conservation of this process in dogs, and it is possible that polarization to IL-17 producing cells could be enhanced even further by blockade of Th1 and Th2 cell differentiation [13]. The production of IL-17 in these cultures was confirmed using a canine-specific IL-17 ELISA (Table 2). In the culture supernatants from unstimulated cells, IL-17 was undetectable in samples from one dog, and present at 0.4 (±0.02) ng/mL in samples from the other. Each of the ConA-stimulated samples from both dogs, including those where IL-2 and TGF-β, or IL-1β, IL-6, and TGF-β were added, contained >8.5 ng/mL of IL-17.

**Table 2:** IL-17 Production by ConA-Stimulated PBMC ^a^.

**Condition**	**Dog-1**	**Dog-2**
	IL-17 (±S. D.; ng/mL)	
Unstimulated	0 (0)	4.5 (0.8)
ConA	8.3 (0.3)	8.4 (1.7)
ConA+TGF-ß+IL-2	8.4 (1.0)	9.8 (0.4)
ConA+TGF-ß+IL-1ß+IL-6	9.5 (2.7)	9.4 (0.3)

^a^ PBMC from two dogs were cultured without stimulation or stimulated by ConA. After 72 h, supernatants were removed and cells were provided with fresh culture media with no additional stimuli, TGF-ß and IL-2, or TGF-ß, IL-1ß, and IL-6 as indicated. Supernatants were recovered 24 h later and IL-17 in each condition was quantified in duplicate with a canine specific IL-17 ELISA. Supernatants were diluted five-fold to ensure values were within the dynamic range of the assay (0–2.0 ng/mL). Values for each sample reflect calibration to the actual concentration.

## 3. Materials and Methods

**Dogs**: Six companion dogs were recruited for this study. Dogs belonged to faculty, students, or staff of the University of Minnesota Veterinary Medical Center (St. Paul, MN, USA). In order to be eligible, dogs had to be healthy based on a complete physical exam, and they had to be older than 3 years of age. Samples were obtained under the supervision of the University of Minnesota Institutional Animal Care and Use Committee (protocol 1101A94713) with informed consent from the owners. Whole blood (7–10 mL) was collected using a standard jugular venipuncture approach and evacuated into sterile tubes containing ethylenediaminetetraacetic acid (EDTA) as an anticoagulant. Dogs did not require hospitalization or special housing for the procedure and they were released back to their owners after a short period of observation to ensure neither bleeding nor hematoma formation were evident at the site of venipuncture.

**Cells and Cell Culture**: Peripheral blood mononuclear cells (PBMCs) were enriched from EDTA-preserved whole blood using a discontinuous Ficoll-Hypaque density gradient [14]. For intracellular IL-17 staining, PBMCs were cultured for 4–6 h in the presence or absence of ConA (2.5 µg/mL, Sigma-Aldrich, St. Louis, MO, USA) with Protein Transport Inhibitor Cocktail reagent (2 µL/mL, eBioscience, San Diego, CA, USA) to prevent protein secretion. For polarization experiments, PBMCs were cultured as above for 3 days, after which fresh media was replenished to the unstimulated cells and ConA-stimulated cells were divided into three groups that received fresh media alone, fresh media supplemented with human recombinant IL-2 (hrIL-2, 10 IU/mL, NCI BMRP, Bethesda, MD, USA) and human TGF-β (5 ng/mL, R&D Systems, Minneapolis, MN, USA), or fresh media supplemented with canine recombinant IL-1β (10 ng/mL, R&D Systems), canine recombinant IL-6 (50 ng/mL, R&D Systems), and TGF-β. Cell-free supernatants were collected 24 hr later to analyze secreted IL-17 using a canine specific IL-17 ELISA, and each culture condition was replenished with media containing the same factors, with Protein Transport Inhibitor Cocktail (2 µL/mL) added for the last 4 h of culture.

**Flow Cytometry**: Cell handling, antibody staining, and flow cytometric analysis were done as previously described [15]. Antibodies were used to identify T cells (anti-CD5 conjugated to phycoerythrin (PE), eBioscience), B cells (anti-CD22 conjugated to allophycocyanin (APC), Abcam, Cambridge, MA, USA), and monocytes (anti-CD14 conjugated to fluorescein isothiocyanate (FITC), AbD Serotec, Raleigh, NC, USA). Conditions were included to enable further separation of T cells based on expression of CD4 (anti-CD4-FITC), CD8 (anti-CD8-APC), and CD25 (anti-CD25-PE), all of which were obtained from eBioscience. Staining with anti-CD45 antibodies (eBioscience) conjugated to PE, APC, and FITC was used for color compensation. Mouse IgG1 and IgG2a antibodies with irrelevant specificities, conjugated to the appropriate fluorochromes (eBioscience) were used as isotype controls. After the initial staining steps, cells were permeabilized using the Foxp3/Transcription Factor Fixation/Permeabilization Concentrate and Diluent kit (eBioscience) for 45 min followed by intracellular staining for FoxP3 (anti-FoxP3-FITC, eBioscience) and for accumulated IL-17 (anti-mouse IL-17 clone eBio17B7) as recommended by the manufacturer [16]. Similar results were obtained in two samples that were stained concurrently using the anti-mouse IL-17 antibody and anti-human IL-17 (clone eBio64DEC17, eBioscience). Multi-parameter flow cytometry was done using the FACSCalibur instrument platform (BD Biosciences, San Jose, CA, USA), and data were analyzed using FlowJo (Treestar, Ashland, OR, USA).

**ELISA**: A canine-specific IL-17 ELISA (R&D Systems) was used following the manufacturer’s recommendations. The concentration of IL-17 in cell-free supernatants was extrapolated from an IL-17 standard curve based on absorbance values obtained from each undiluted (“neat”) sample and from each sample diluted five-fold.

## 4. Conclusions

In summary, our results show that mitogenic stimulation leads to IL-17 production by both CD4 and CD8 cells, and that anti-inflammatory or pro-inflammatory cytokine signals, respectively, promote expansion of Tregs or Th17 cells. Tc17 cells show species differences: in humans, but not in mice, these cells arise from a unique subset of CD161^+^ precursors [5]. A similar subset remains to be defined in dogs, so it is not clear if Tc17 cells in this species would be more similar to human or mouse. Considering the importance of IL-17 in autoimmune disease, inflammation and cancer, continued research in this area will help elucidate the role that these important adaptive immune cells play, potentially enabling better treatment options for these conditions.
